# Nonoperative treatment of avulsion fracture of the anterior tibia with proximal fibular fracture: A case report

**DOI:** 10.3389/fsurg.2022.959008

**Published:** 2023-01-06

**Authors:** Jian Yu, Chao Zhang, Xu Wang, Xin Ma, Jiazhang Huang

**Affiliations:** Department of Orthopedics, Huashan Hospital, Fudan University, Shanghai, China

**Keywords:** avulsion fracture of the anterior tibia, proximal fibular fracture, nonoperative management, Maisonneuve fracture, ankle instability

## Abstract

**Background:**

Avulsion fracture of the anterior tibia with proximal fibular fracture commonly occurs in Maisonneuve fracture, which generally involves ankle instability and requires surgical correction. Nonoperative treatment of this type of fracture has been rarely reported in the literature.

**Case presentation:**

A 48-year-old male reported pain in the lateral part of his left lower leg and ankle during a badminton play. Physical examination revealed tenderness and swelling of the lateral aspects of the left ankle, as well as the proximal aspect of the fibula. Preoperative plain x-ray image, computed tomography, and magnetic resonance imaging revealed an avulsion fracture of the anterior tibia by the anterior inferior tibiofibular ligament without medial and posterior fracture, rupture of the deltoid ligament, or interosseous membrane. Nonoperative management was performed and successful recovery was observed at a 6-month follow-up.

**Conclusions:**

Nonoperative management can be a better option for some variations of Maisonneuve fracture with a stable ankle joint. The selection of treatment options should be based on careful examination and radiological evaluation of the ankle.

## Introduction

Avulsion fracture of the anterior tibia with proximal fibular fracture commonly occurs in Maisonneuve fracture, which generally involves ankle instability and requires surgical correction. Nonoperative treatment of this type of fracture has been rarely reported in the literature.

In this article, we report a case only involving proximal fibular fracture and avulsion fracture of the anterior tibia without disrupting the medial or posterior structures. The patient was treated with nonoperative management and successfully returned to his sport and daily life activity. To our knowledge, this variant of a Maisonneuve fracture has not been reported. The patient was informed that data concerning this case would be submitted for publication. We present the following case in accordance with the CARE reporting checklist.

## Case report

A 48-year-old male reported pain in the lateral part of his left lower leg and ankle during a badminton play. He was injured when he attempted to receive a high serve. The shuttlecock came to his left, so he stepped his right foot to the left first and jump to hit it. However, he felt pain at the left lower extremity when his left foot landed on the ground and immediately lost the ability to continue the play. He denied a direct impact injury to the proximal end of the left leg and confirmed no history of trauma or surgical treatment on the lower extremity.

Physical examination revealed no obvious deformity and intact skin of the left leg with mild tenderness upon palpation of the lateral malleolus, as well as the proximal aspect of the fibula. Medial or posterior tenderness was not presented. The initial motions of the ankle joint were limited and there was no distal neurovascular deficit. There were no other skeletal injuries and the systemic examination was unremarkable.

**Figure 1:**
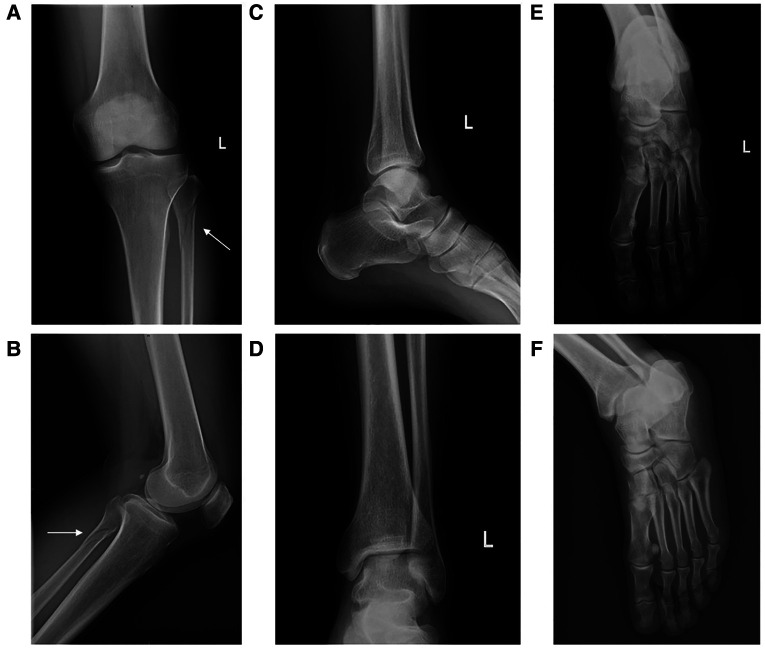
The initial x-ray images of the proximal fibula (**A,B**), the ankle (**C,D**), and the foot (**E,F**).

Plain radiographs demonstrated a spinal fracture at the proximal fibula with no displacement of fragments. Evidence of a medial, lateral, or posterior malleolar fracture or other defects was not clear (Figure 1). Talar shift or widening of the ankle mortise was not noted.

**Figure 2:**
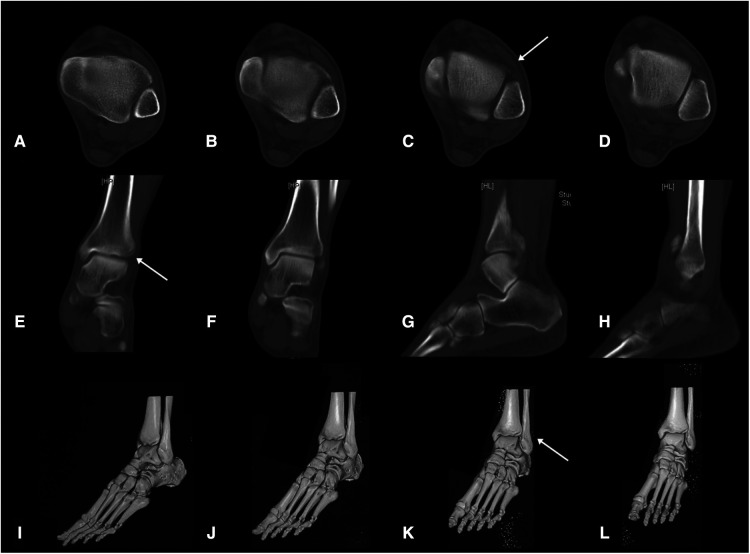
The initial transverse view (**A–D**), coronal view (**E,F**), and sagittal view (**G,H**) CT images of the ankle, and 3D reconstruction image of the foot and ankle (**I–L**).

**Figure 3:**
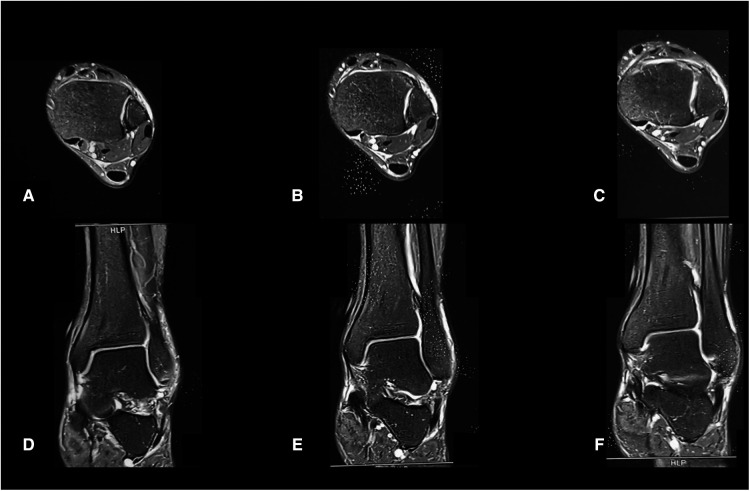
The initial transverse view (**A–C**) and coronal view (**D–F**) MRI images of the ankle.

Computed tomography (CT) images of the ankle showed an avulsion fracture of the anterior tibia by the anterior inferior tibiofibular ligament (AITFL) with a nondisplaced bony fragment (Figure 2). However, magnetic resonance images suggested no injury of the posterior inferior tibiofibular ligament, interosseous tibiofibular ligament, deltoid ligament, and both the medial and lateral malleoli (Figure 3).

**Figure 4:**
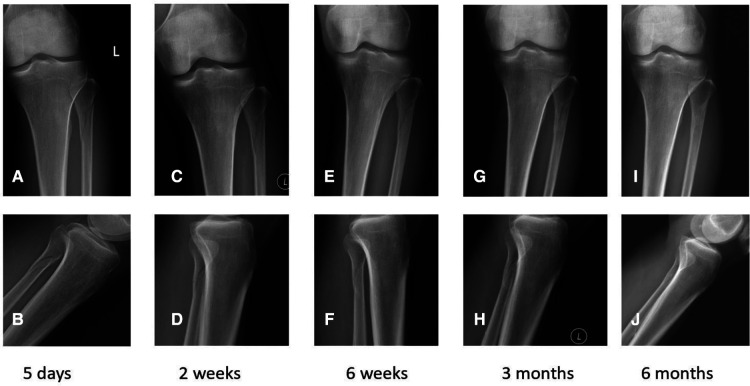
Frontal (**A, C, E, G, I**) and lateral (**B, D, F, H, J**) view CT images of the proximal tibia (5 days, 2 weeks, 6 weeks, 3 months, and 6 months after the injury).

Considering the ankle was stable, we decided to perform nonoperative management. The injury was treated with rest, ice, and elevation. Immobilization of the left lower leg was done with a short leg cast for 6 weeks. Six weeks later, the cast was removed. After that, gradual resumption of ankle range of motion, leg strengthening exercises, and regular activities began. The patient initially walked with hand support. Three months after the injury, the patient started to walk with full body weight. The patient presented free of pain, good ankle motion, and functional recovery during follow-ups at 6 weeks, 3 months, and 6 months after the injury. Plain radiographs showed successful recovery of the fracture at the proximal fibula (Figure 4). At the final follow-up, his American Orthopaedic Foot and Ankle Society ankle-hindfoot (AOFAS) score was 100 out of 100 total points, and he returned to his regular sport, badminton.

## Discussion

In this study, we report an unusual type of injury only involving proximal fibular fracture and avulsion fracture of the anterior tibia with intact medial and posterior structures. We treated with nonoperative management and the patient received a good recovery at the 6-month follow-up.

The primary concern about this case is its injury mechanism. The patient has been through an acute high-energy injury with rotational force during a badminton play and he denied a direct impact injury to the proximal end of his left leg. X-ray images showed a proximal quarter fibular fracture and intact medial and posterior structure of the ankle. CT images found that avulsion fractures occurred at one of the insertion sites of the AITFL, which was the anterior tibial tuberosity. Both isolated avulsion fracture of the anterior tibia and proximal fibular fracture are rare in adults (1–3). The combination of these two injuries enabled us to first consider the diagnosis of Maisonneuve fracture. Axial and external rotational forces were transmitted from the bottom-up, traveled through ankle syndesmosis and the medial and posterior malleoli, then up toward the interosseous membrane, and finally exited at the proximal fibula *via* fracture. However, the intact medial and posterior structure of the tibia indicated its specialty.

Maisonneuve fracture is a unique fracture pattern of ankle fracture often associated with the fracture of the proximal 1/3 of the fibula and involves damage to the medial structure (medial malleolus and deltoid ligament) and ankle syndesmosis. It accounts for about 5% of the ankle fracture estimated by Pankovich (3), and this type of fracture is often missed diagnosed due to no examination of the proximal fibula (4). The injury mechanism of this type of ankle fracture is not crystal clear. It was generally considered as Lauge-Hansen pronation-external rotation type fracture (5–7), Danis and Weber type C fracture, or AO type C3 fracture.

In Lauge-Hansen classification (5), stage I pronation-external rotation injuries represent injury to the medial structures (medial malleolus or deltoid ligament), stage II is the rupture of the anterior ankle syndesmosis and interosseous membrane, stage III presents the fibula fracture above the level of the syndesmosis, and stage IV has the rupture of the posterior ankle syndesmosis or fracture of the posterior malleolus. Maisonneuve fracture was considered to occur in stage III of pronation-external rotation injuries. However, this mechanism does not explain cases with intact medial structure.

Pankovich (3) described five stages in the injury mechanism of Maisonneuve fracture: (i) rupture of the anterior tibiofibular ligament or avulsion fracture of one of its bone insertions, either one being associated with rupture of the interosseous ligament; (ii) fracture of the posterior tubercle or rupture of the posterior tibiofibular ligament; (iii) rupture of the anteromedial joint capsule or avulsion fracture of one of its bone insertions; (iv) fracture of the proximal part of the fibula; and (v) rupture of the deltoid ligament or fracture of the medial malleolus. This sequence corresponds to the supination-external rotation mechanism as proposed by Lauge-Hansen and can explain why the medial structures are left intact in Maisonneuve fracture. However, this mechanism cannot well explain the case with an intact posterior structure.

Most of the Maisonneuve fracture requires surgical management due to unstable ankle mortise or the disruption of ankle syndesmosis (8–14). Nonoperative management is rarely reported in the literature (15). Pankovich (3) treated the Maisonneuve fracture nonoperatively in cases without rupture of the deltoid ligament, interosseous ligaments, or medial malleolus fracture. Merrill (16) suggested that these are often more stable than generally assumed and recommended nonoperative treatment for injuries with only partial disruption of the syndesmosis and an intact posterior hinge (namely, the posterior inferior tibiofibular syndesmosis and transverse tibiofibular ligaments). Lock et al. (4) recommended long leg casting for 6–12 weeks if no medial malleolar fracture is present and the medial joint line was not widened. Charopoulos et al. (15) reported a rare injury involving fracture of the proximal fibula in association with posterior malleolar fracture and disruption of the anterior inferior tibiofibular ligament, without disruption of the deltoid ligament or fracture of the medial malleolus. Nonoperative treatment of the patient was successful.

In the current case, our choice of nonoperative treatment for the patient was based on the fact that the majority of the stabilizers of ankle mortise appeared to remain undamaged, including the deltoid ligament, posterior inferior tibiofibular ligament, and interosseous tibiofibular ligament. The spaces of the distal tibiotalar, medial, and lateral malleolar articular surfaces were not widened. Although avulsion fractures were observed at the anterior tibia, the benefit of surgical treatment may be limited. A small shell at the tibia cannot be easily reduced, which is too small for any fixation implant. Also, it is not necessary to surgically fix the nondisplaced fracture of the distal tibia and the proximal fibula. Implanting a metal plate at the proximal fibula may injure the common peroneal nerve.

A weight-bearing anteroposterior (AP) view, a gravity stress view, or external rotation stress view of the x-ray scan can be valuable for the evaluation of inferior syndesmotic instability (17–19). However, ankle magnetic resonance imaging showed no obvious tear of the posterior inferior tibiofibular ligament and interosseous tibiofibular ligament, and ankle syndesmotic diastasis was not presented in plain radiographs. We concluded that the ankle was stable with the consideration of the clinical examination and imaging data of this patient. Therefore, we did not additionally schedule a weight-bearing or stress view of the x-ray scan, which may induce ankle pain, and it is one of the limitations of this case report.

## Conclusion

Not all Maisonneuve fractures require surgical treatment and patients with a special type of this fracture can present with good recovery after nonoperative management as described in this case report. The selection of treatment options should be based on careful examination and radiological evaluation. Disruption of the tibiofibular and proximal fibular fractures with intact medial and posterior malleolus may have a unique injury mechanism that cannot be fully explained by the Lauge-Hanson classification of the ankle fracture. Future biomechanical studies should explore the injury mechanism behind this type of fracture.

## Data Availability

The original contributions presented in the study are included in the article/Supplementary Material, further inquiries can be directed to the corresponding authors.
